# Sex differences in hypertension among people living with HIV after initiation of antiretroviral therapy

**DOI:** 10.3389/fcvm.2022.1006789

**Published:** 2022-11-17

**Authors:** Sepiso K. Masenga, Joreen P. Povia, Katongo H. Mutengo, Benson M. Hamooya, Selestine Nzala, Douglas C. Heimburger, Sody M. Munsaka, Fernando Elijovich, Kaushik P. Patel, Annet Kirabo

**Affiliations:** ^1^HAND Research Group, School of Medicine and Health Sciences, Mulungushi University, Livingstone, Zambia; ^2^School of Medicine, University of Zambia, Lusaka, Zambia; ^3^Department of Medicine, Vanderbilt Institute for Global Health, Vanderbilt University Medical Center, Nashville, TN, United States; ^4^Department of Biomedical Sciences, School of Health Sciences, University of Zambia, Lusaka, Zambia; ^5^Department of Cellular and Integrative Physiology, University of Nebraska Medical Center, Omaha, NE, United States

**Keywords:** incident hypertension, HIV, dolutegravir, protease inhibitors, blood pressure, lopinavir, sex differences

## Abstract

**Background:**

Hypertension is common in people living with HIV (PLWH) on antiretroviral therapy (ART). In the general population and in experimental animal models, the incidence of hypertension is greater in males than in females, especially during the premenopausal period. However, it is not known whether there are sex differences in hypertension associated with HIV and ART, and the factors contributing to incident hypertension among PLWH have not been well characterized. In this study, we aimed to determine the time course, sex differences and factors associated with incident hypertension in PLWH initiating ART.

**Methods and results:**

We conducted a retrospective study in which we used programmatic data from the ART registry to identify sex differences in the determinants of incident hypertension among PLWH initiating the ART regimen from Livingstone University Teaching Hospital in Zambia and followed for 8 years. Males developed hypertension earlier, 2 years after initiating ART, compared to 6 years in females. In multivariable analysis, increasing age, baseline systolic blood pressure and baseline mean arterial pressure (MAP) were associated with increased risk for developing incident hypertension. Also, participants who switched to the integrase strand transfer inhibitor, dolutegravir (DTG) or the protease inhibitor, lopinavir boosted with ritonavir were 2 and 3 times more likely to develop hypertension when compared to those on non-nucleoside reverse transcriptase inhibitors (NNRTIs). However, these relationships were abrogated by sex, as self-reported male sex was the major contributor in predicting incident hypertension. While none of the factors remained significantly associated with incident hypertension upon multivariate analysis among females, body mass index (BMI), and use of protease inhibitors remained strongly associated with hypertension among males.

**Conclusion:**

Our results indicate that the use of protease inhibitors and BMI are important predictors of incident hypertension among males. Thus, blood pressure and BMI should be closely monitored, particularly in males living with HIV on protease inhibitors. In addition, identifying specific factors that protect females from developing hypertension early is important but remains to be determined.

## Introduction

The susceptibility to non-communicable diseases such as hypertension is a rising challenge facing people living with HIV (PLWH), especially in low-income countries (1). The estimated prevalence of hypertension among PLWH in sub-Saharan Africa is up to 50% (2). PLWH who develop hypertension are at increased risk for cardiovascular disease, kidney injury, and death (3). The development of hypertension in PLWH is associated with several factors including the use of certain antiretroviral therapies (ART), increasing age, dietary salt, body mass index (BMI), duration of ART, and immune cell activation (4–6).

Emerging data from recent studies have demonstrated a higher risk of hypertension in males when compared to age−matched, pre−menopausal females (7, 8). However, the different factors involved in potentially explaining how sex contributes to hypertension are not entirely clear (9). In PLWH, there is limited data on sex differences in hypertension. Also, despite the shift to ART regimens based on integrase strand transfer inhibitors (INSTIs) across Sub-Saharan Africa, there is limited information with regard to their association with hypertension in this region. The use of INSTIs has been associated with weight gain and thus may contribute to an increased risk of developing hypertension (10).

The time course and contributing factors for PLWH to develop hypertension after initiating ART are unknown, especially in sub-Saharan Africa where the burden of hypertension among PLWH is highest (11). Data on predictors of incident hypertension and sex differences in PLWH from sub-Saharan African populations including Zambia are limited and need to be examined. Thus, the goal of this study was to determine the time course, sex differences, and factors associated with incident hypertension in a Zambian population of PLWH following initiation of ART.

## Materials and methods

### Study population

**FIGURE 1:**
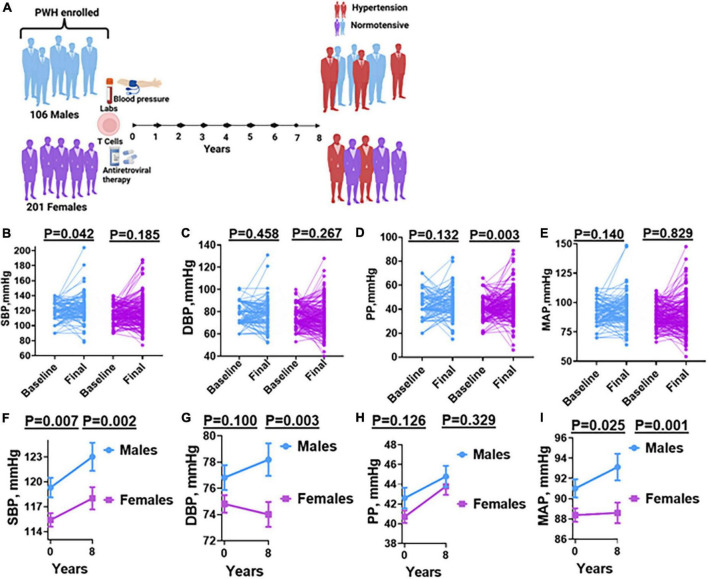
Blood pressure measurements before and after antiretroviral therapy in males and females. **(A)** Schematic of experimental design. **(B)** SBP, **(C)** DBP, **(D)** PP, **(E)** MAP: shows changes in individual parameters at baseline and final (after antiretroviral therapy) in males and females. **(F)** SBP, **(G)** DBP, **(H)** PP, **(I)** MAP: shows composite means of each parameter at baseline and final (after antiretroviral therapy) in males and females. Wilcoxon matched-pairs signed rank test and paired *t*-test was used to determine the *P*-values. SBP, systolic blood pressure; DBP, diastolic blood pressure; PP, pulse pressure; MAP, mean arterial pressure.

We conducted a retrospective cohort study in which we used programmatic data from the ART registry to identify 307 PLWH from Livingstone University Teaching Hospital in Zambia (Figure 1A).

### Inclusion and exclusion criteria

We included PLWH adults aged above 18 years who were normotensive when initiating ART, and examined their clinical records retrospectively for 8 years following initiation of treatment. The follow-up time was uniform for all the participants such that only those who were still enrolled in the ART clinic until the 8th year were included. We excluded any patient who was hypertensive or had a single systolic or diastolic blood pressure (SBP/DBP) ≥ 140/90 mmHg before ART and those with missing blood pressure measurements in any given quarter of each year.

### Ethics approval and consent to participate

This study was approved by the University of Zambia Biomedical Research Ethics committee (IRB00001131 of IORG0000774) and the National Health Research Ethics Board (NHREB) under Reference number 981-2020.

### Data collection

We reviewed data for PLWH who were enrolled in the ART clinic between 2005 and 2012 and identified every 10th patient from the file who passed our eligibility criteria. As is common with secondary data, we found a lot of missing results and had to constantly shift the sampling frame for additional participants. Based on our inclusion criteria were only those who were still enrolled in the ART clinic until the 8th year were included, the follow-up rate was therefore 100%. We used Research Electronic Data Capture (REDCap) to collect and manage data (12, 13). We collected the following sociodemographic and clinical characteristics of participants: age, sex, ART regimen profile, BMI, CD4 count, and serum creatinine. BMI, CD4 count and creatinine were monitored both at baseline (the day of ART initiation) and at the end of the 8 years of follow-up. All participants were initiated on non-nucleoside reverse transcriptase inhibitors (NNRTIs) at baseline. However, some participants’ initial regimen was changed in the course of treatment to either an INSTI or protease inhibitor (PI) while others were maintained on NNRTIs up to the final time point. All blood pressure readings for each year were averaged to compute annual blood pressure readings.

### Measurement of blood pressure

Blood pressure measurements were made with an automated sphygmomanometer. The routine protocol for accurate blood pressure measurements in the clinic includes the following: the participants must be seated, with their arms at heart level and their feet flat on the floor and their back supported by a chair (14). For diagnosis of hypertension, we used cut-offs for SBP/DBP of ≥ 140/90 mmHg on more than 2 consecutive occasions. We further confirmed this diagnosis by reviewing patient files to determine if they were prescribed antihypertensive medication after hypertension defining BPs. Most participants were later on prescribed two antihypertensive drugs, a calcium channel blocker (amlodipine or nifedipine) and an angiotensin-converting enzyme inhibitor (ACE) (enalapril or losartan) or a diuretic (furosemide or Moduretic). Hypertension-free survival time in this study is defined as the time in years until the diagnosis of hypertension was made.

### Statistical analysis

We used statistical package for social sciences (SPSS) and Stat Crunch for data analysis. To compare proportional distributions of each categorical characteristic with hypertension status we used Chi-square. Adjusted standardized ratios were computed to determine significant proportions in the cells. Shapiro Wilk test was used to test for normality of the data. Mann-Whitney was used to compare medians of two continuous variables. Comparison of blood pressure, creatinine, CD4 count, and BMI were carried out with the Wilcoxon matched-pairs signed-rank test contrasting median values between the baseline and the final. One-sample proportion summary hypothesis test with the Standard-Wald method was used to determine the time course when the majority of hypertensive persons would be diagnosed and to compute 95% confidence limits of proportions. Hypertension-free survival time was compared between groups using Kaplan Meier curves. To test for statistical significance, log-rank test was used. Univariable and multivariable Cox proportional hazard regression were used to investigate the effects of several variables on the time course of hypertension diagnosis. Only variables with *p*-values ≤ 0.02 were added to the final model to avoid over-adjustment. Statistical significance was assessed using 95% confidence interval (CI) and 5% level of significance.

## Results

### Characteristics of the study population

We studied 307 PLWH with a median age of 47 years and 65.5% female (Table 1). The incidence of hypertension was 11.7%. Among those who developed hypertension, about 33% became hypertensive in the first year after initiating ART, and the proportion doubled (69%) by the end of year 2, representing more than half of the total population, *p* = 0.009 (Table 2). Although all participants were initiated on NNRTIs, efavirenz- or nevirapine-based regimens, by the end of the follow-up, the majority (*n* = 188, 61.2%) remained on NNRTIs while 22.1% (*n* = 68) and 16.6% (*n* = 51) had switched to an INSTI dolutegravir (DTG) and protease inhibitors (PI) lopinavir boosted with ritonavir, respectively (Table 1). The majority (84.9%) of participants were virally suppressed (Table 1).

**TABLE 1:** Study characteristics and factors associated with incident hypertension.

Variable	Total, *n* (%) or median (interquartile range)	Hypertension status, *n (%*) or median (interquartile range)		*p*
		
		Hypertensive, *36 (11.7*)	Normotensive, *271 (88.3*)	
**Age**	47 (42, 54)	54 (48, 60)	46 (41, 52)	< 0.001**
**Sex**				
*Male*	106 (34.5)	13 (36.1)	93 (34.3)	0.83
*Female*	201 (65.5)	23 (63.9)	178 (65.7)	
**Antiretroviral therapy**				0.004***
*Nucleoside/non-nucleoside*	188 (61.2)	13 (36.1)	175 (64.6)	
*Dolutegravir-based*	68 (22.1)	14 (38.9)	54 (19.9)	
*Protease inhibitors*	51 (16.6)	9 (25.0)	42 (15.5)	
**Switched ART**				
*No*		1 (2.8)	53 (19.6)	0.009*
*Yes*		35 (97.2)	218 (80.4)	
**BMI,** *Kg/m^2^*	22.5 (19.6, 26.5)	25.1 (20.8, 29.5)	22.2 (19.5, 25.8)	0.007**
***CD4 count**, cells/*μ*L*	462 (335, 621)	463 (309, 594)	474 (336, 640)	0.25**
***Viral suppression*** **< *1,000 copies/ml, n* = *239***				
*Yes*	203 (84.9)	30 (100.0)	173 (82.8)	0.011***
*No*	36 (15.1)	0 (0.0)	36 (17.2)	
***Creatinine***, μ*mol/L*	74 (59, 91)	86 (67, 101)	75 (58, 91)	0.029**

ART, antiretroviral therapy; BMI, body mass index; Column percentage; *Fisher’s exact test; **Mann-Whitney tests; ***Chi-square. Bold values indicate *p*-values <0.05.

**TABLE 2:** Cumulative proportion of participants who developed hypertension at each time interval.

Time taken (years) to hypertension diagnosis	Developed hypertension, *n* = 36 (proportion)	95% CI	*P*
**1**	12 (0.33)	0.17–0.48	0.97
**2**	25 (0.69)	0.54–0.84	**0.009**
**3**	26 (0.72)	0.57–0.86	**0.003**
**4**	27 (0.75)	0.60–0.89	**0.001**
**5**	29 (0.81)	0.67–0.93	**0.0001**
**6**	33 (0.92)	0.82–1.00	** < 0.0001**
**7**	35 (0.97)	0.91–1.02	** < 0.0001**
**8**	36 (1.0)	NA	** < 0.0001**

Proportions estimated with one sample proportion summary hypothesis testing. CI, confidence interval; NA, not applicable. Bold values indicate *p*-values <0.05.

### Sex differences in blood pressure changes following initiation of antiretroviral therapy in people living with HIV

To determine sex differences in blood pressure changes following initiation of ART in PLWH, we measured blood pressure parameters including SBP, DBP, mean arterial pressure (MAP), and pulse pressure (PP) among self-identified male and female participants both at baseline and after 8 years of follow up (Figure 1A). We found that among males and females, there was variability in how their individual SBP, DBP, PP, and MAP changed from baseline to 8 years (Figures 1B–E). A pair-wise analysis indicated a significant increase in SBP only among males (Figure 1B). There was no significant increase in DBP among both males and females (Figure 1C). Interestingly, we found that the increase in PP was significant only among females when compared to males (Figure 1D). There were no significant increases in MAP among both males and females (Figure 1E). On average, we found that males had a significantly higher SBP when compared to females at baseline which significantly increase after 8 years of follow-up when compared to females (Figure 1F). Although not statistically significant, the DBP in males tended to be higher, which tended to increase over time when compared to females whose DBP tended to paradoxically decrease resulting in a significantly higher DBP in males at 8 years of follow-up (Figure 1G). There were no significant differences in both baselines and 8 years of follow-up average PP among both males and females (Figure 1H). However, we found that males had significantly higher average MAP than females both at baseline and at the end of 8 years of follow-up (Figure 1I).

**FIGURE 2:**
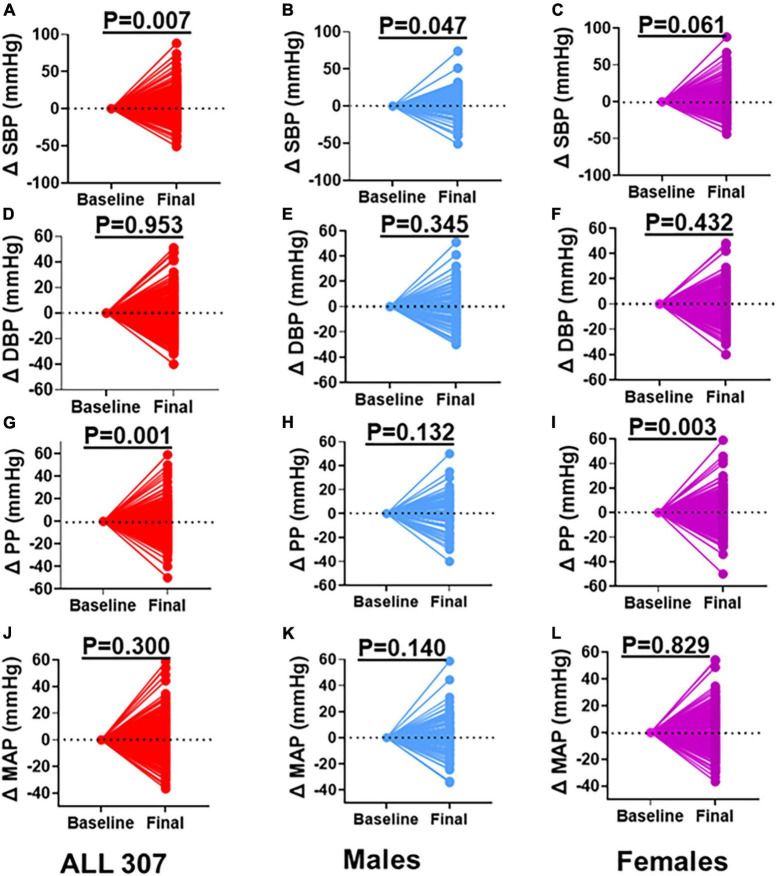
Changes in Blood Pressure between Baseline and Final. **(A)** SBP all participants, **(B)** SBP males, **(C)** SBP females, **(D)** DBP all participants, **(E)** DBP males, **(F)** DBP females, **(G)** PP all participants, **(H)** PP males, **(I)** PP females, **(J)** MAP all participants, **(K)** MAP males, **(L)** MAP females: baseline values were zeroed and differences between final and baseline (delta) compared. Paired *t*-test was used to determine the *P*-values. SBP, systolic blood pressure; DBP, diastolic blood pressure; PP, pulse pressure; MAP, mean arterial pressure.

To determine the degree and variability of the blood pressure changes, we normalized the changes to the baseline. We found that on an individual basis, some participants including males and females exhibited increases, some maintained, and others exhibited blood pressure decreases from baseline to 8-year follow-ups (Figure 2). We found a significant increase in SBP among all participants combined (Figure 2A). This increase in SBP remained significant when we analyzed only men (Figure 2B) but not among women (Figure 2C). However, the change in DBP from baseline was not significant in either all participants combined (Figure 2D), males (Figure 2E) or females (Figure 2F). Interestingly, the PP significantly increased among all participants from baseline to the 8-year follow-ups (Figure 2G). However, this increase was not significant among men (Figure 2H) but was significant among women (Figure 2I). The changes in MAP from baseline to 8-year follow-up was not significant in either all participants (Figure 2J), men (Figure 2K) or females (Figure 2L).

**FIGURE 3:**
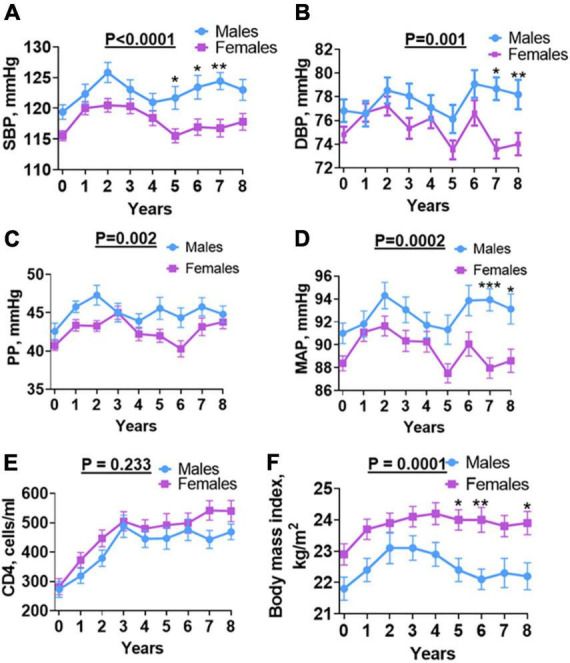
Sex differences in BP, CD4, and BMI over time. **(A)** SBP, **(B)** DBP, **(C)** PP, **(D)** MAP, **(E)** CD4 absolute count and **(F)** body mass index: shows changes in individual parameters each year for 8 years from baseline (before antiretroviral therapy) until final (after antiretroviral therapy) for males and females. SBP, systolic blood pressure; DBP, diastolic blood pressure; PP, pulse pressure; MAP, mean arterial pressure; Welch’s test was used to compare means between males and females. Asterisks indicate a time point with significant differences in blood pressure between males and females. *p < 0.05; **p < 0.01; ***p < 0.001.

To determine overall annual blood pressure changes over time among males and females and determine the contributing factors, we performed a two-way ANOVA analysis on SBP, DBP, PP, and MAP with a *post hoc*, Šídák’s multiple comparisons test. We found that males had significantly higher SBP at baseline, which increased progressively to years 1 and 2, but decreased at years 3 and 4 and then steadily increased in years 5, 6, and 7, but slightly decreased at year 8 of follow-up. The females showed a similar pattern of exhibiting increases in SBP from baseline to year 2, followed by a steady decrease in years 3, 4, and 5, and remained steady in years 6, 7, and 8 (Figure 3A). A similar pattern was observed among men and women for DBP and males exhibited significant increases in DBP only in years 7 and 8 of follow-up (Figure 3B). The PP was significantly higher in males than females and exhibited a pattern of the initial increase from baseline in the first 2 years followed by a decrease as observed for SBP (Figure 3C). The MAP also showed a similar pattern and was significantly higher in men at baseline and years 7 and 8 of follow-up (Figure 3D). In univariate analysis, baseline MAP, SBP, and DBP were associated with increased risk for incident hypertension (Table 3). In multivariable analysis, baseline MAP and SBP remained significantly associated with incident hypertension.

**TABLE 3:** Predictors of incident hypertension in persons with HIV.

Variable	Univariable analysis		Multivariable analysis	
		
	HR (95% CI)	*p*	aHR (95% CI)	*p*
**Age,** *median years (IQR*)	1.06 (1.02–1.09)	**< 0.001**	1.04 (1.01–1.08)	**0.013**
**Sex**				
*Female*	1			
*Male*	1.04 (0.74–1.47)	0.79		
**ART regimen**				
*NNRTI/NRTI*	1		1	
*Integrase (DTG*)	3.15 (1.48–6.70)	**0.003**	2.33 (1.07–5.05)	**0.032**
*Protease (LPV/r) inhibitor*	2.70 (1.15–6.33)	**0.022**	3.27 (1.39–7.71)	**0.007**
***Baseline MAP,** mmHg*	1.06 (1.02–1.09)	**0.001**	1.05 (1.01–1.09)	**0.008**
***Baseline SBP,** mmHg*	1.05 (1.02–1.08)	**0.001**	1.04 (1.00–1.09)	**0.027**
***Baseline DBP,** mmHg*	1.04 (1.01–1.08)	**0.006**	1.01 (0.97–1.05)	0.620
**Baseline BMI,** *Kg/m^2^*	1.05 (0.98–1.12)	0.11		
**Final BMI,** *Kg/m^2^*	1.06 (1.01–1.13)	**0.020**	1.04 (0.98–1.10)	0.170
***CD4 at initiation,** cells/*μ*L*	1.00 (1.00–1.00)	0.15		
***Final CD4 Count,** cells/*μ*L*	1.00 (1.00–1.00)	0.07		
***Viral suppression*** **< *1,000 copies/ml***				
*Yes*	1			
*No*	0.19 (0.02–1.64)	0.13		
***Baseline creatinine,*** μ*mol/L*	1.00 (0.99–1.01)	0.12		
***Final creatinine,*** μ*mol/L*	1.00 (0.99–1.00)	0.51		

ART, antiretroviral therapy; CI, confidence interval; HR, hazard ratio; aHR, adjusted hazard ratio; NNRTI, Non-Nucleoside Reverse Transcriptase Inhibitor; NRTI, Nucleoside Reverse Transcriptase Inhibitor; DTG, dolutegravir; LPV/r, ritonavir boosted lopinavir; IQR, interquartile range; MAP, mean arterial pressure; SBP, systolic blood pressure; DBP, diastolic blood pressure; BMI, body mass index; *p*-values of 0.02 or less were included in the multivariable model. Bold values indicate *p*-values <0.05.

### Effect of CD4 count on development of incident hypertension following initiation of antiretroviral therapy in people living with HIV

HIV infection is associated with a depletion of CD4 T cells. Initiation of ART results in reconstitution of CD4 T cells and this has been associated with blood pressure elevation in PLWH in the first 2 years of follow-up (15). To determine if CD4 T cell counts play a role in sex differences observed in blood pressure, we analyzed numbers of CD4 T cells at baseline and annually during the 8 years of follow-up (Figure 3E). After initiating ART, CD4 count increased for both male and female participants (Figure 3E). The median CD4 T cell count was comparable between males and females at all-time points during the follow-up. We found no significant difference in baseline CD4 T cell counts among men and women. The CD4 T cell counts steadily increased in the first 3 years following the initiation of ART in a manner that was similar among men and women. The CD4 T cell counts plateaued among men and women during years 3–6. However, we observed an increase in CD4 T cell counts during years 7 and 8 of follow-up among women but not among men (Figure 3E). However, this increase was not statistically and significantly different from that of men. Baseline CD4 predicted incident hypertension in females (Table 4) and not in males (Table 5) on univariate analysis but did not remain a significant predictor of hypertension on multivariate analysis.

**TABLE 4:** Predictors of incident hypertension in females with HIV.

Variable	Univariable analysis		Multivariable analysis	
		
	HR (95% CI)	*p*	aHR (95% CI)	*p*
**Age,** *median years (IQR*)	1.07 (1.02–1.13)	**0.002**	1.05 (0.99–1.11)	0.07
**ART regimen**				
*NNRTI/NRTI*	1		1	
*Integrase (DTG*)	4.61 (1.69–12.55)	**0.003**	3.33 (0.91–12.09)	0.06
*Protease (LPV/r) inhibitor*	1.58 (0.46–5.40)	0.46	2.50 (0.51–12.05)	0.25
***Baseline SBP,** mmHg*	1.07 (1.02–1.11)	**0.002**	1.05 (0.98–1.13)	0.10
***Baseline DBP,** mmHg*	1.07 (1.02–1.12)	**0.004**	1.03 (0.96–1.10)	0.37
**Baseline BMI,** *Kg/m^2^*	1.01 (0.93–1.11)	0.69	0.92 (0.82–1.03)	0.16
**Baseline CD4,** *cells/*μ*L*	1.00 (1.00–1.01)	**0.04**	1.00 (0.99–1.01)	0.14
**Baseline creatinine**, μ*mol/l*	1.01 (0.99–1.02)	0.14	1.01 (0.99–1.02)	0.20

ART, antiretroviral therapy; CI, confidence interval; HR, hazard ratio; aHR, adjusted hazard ratio; NNRTI, Non-Nucleoside Reverse Transcriptase Inhibitor; NRTI, Nucleoside Reverse Transcriptase Inhibitor; DTG, dolutegravir; LPV/r, ritonavir-boosted lopinavir; IQR, interquartile range; MAP, mean arterial pressure; SBP, systolic blood pressure; DBP, diastolic blood pressure; BMI, body mass index; *p*-values of 0.02 or less were included in the multivariable model. Bold values indicate *p*-values <0.05.

**TABLE 5:** Predictors of incident hypertension in males with HIV.

Variable	Univariable analysis		Multivariable analysis	
		
	HR (95% CI)	*p*	aHR (95% CI)	*p*
**Age,** *median years (IQR*)	1.05 (0.99–1.11)	0.10	1.01 (0.93–1.09)	0.76
**ART regimen**				
*NNRTI/NRTI*	1		1	
*Integrase (DTG*)	2.88 (0.64–12.95)	0.16	1.56 (0.24–9.81)	0.63
*Protease (LPV/r) inhibitor*	7.87 (1.63–37.95)	**0.01**	16.45 (1.72–156.64)	**0.01**
***Baseline SBP,** mmHg*	1.04 (0.99–1.10)	0.10	1.02 (0.93–1.10)	0.63
***Baseline DBP,** mmHg*	1.02 (0.96–1.08)	0.41	1.05 (0.95–1.16)	0.31
**Baseline BMI,** *Kg/m^2^*	1.20 (1.03–1.40)	**0.01**	1.30 (1.06–1.59)	**0.01**
**Baseline CD4,** *cells/*μ*L*	0.99 (0.99–1.00)	0.13	0.99 (0.98–1.00)	0.33
**Baseline creatinine**, μ*mol/l*	1.00 (0.97–1.02)	0.91	1.0 (0.96–1.03)	0.98

ART, antiretroviral therapy; CI, confidence interval; HR, hazard ratio; aHR, adjusted hazard ratio; NNRTI, Non-Nucleoside Reverse Transcriptase Inhibitor; NRTI, Nucleoside Reverse Transcriptase Inhibitor; DTG, dolutegravir; LPV/r, ritonavir-boosted lopinavir; IQR, interquartile range; MAP, mean arterial pressure; SBP, systolic blood pressure; DBP, diastolic blood pressure; BMI, body mass index; *p*-values of 0.02 or less were included in the multivariable model. Bold values indicate *p*-values <0.05.

### Sex differences in body mass index following initiation of antiretroviral therapy in people living with HIV and impact on incident hypertension

Obesity and overweight have been associated with hypertension in the general population (16). To determine if increased BMI was associated with increased hypertension following initiation of ART among men and women with HIV, we analyzed BMI both at baseline and annually for the 8 year follow-up period (Figure 3F). We found that contrary to blood pressure, BMI was higher among women at baseline as well as annually throughout the 8-year follow-up period when compared to men, except at year 2. Among both men and women, BMI increased from baseline reaching a plateau at year 2–8 in women but decreased progressively from year 2 to 6, and then plateaued at year 7 and 8 (Figure 3F). The baseline BMI was 22.5 Kg/m^2^ (Table 1). The final BMI was significantly higher in PLWH who developed hypertension compared to PLWH who did not develop hypertension (25.1 Kg/m^2^ vs. 22.2 Kg/m^2^, *p* = 0.007). In univariate but not multivariable analyses, BMI at the end of follow-up was associated with an increased risk for incident hypertension (Table 3). BMI of females was higher than that of males throughout the follow-up period (Figure 3F).

### Age and incident hypertension following initiation of antiretroviral therapy in people living with HIV and impact of sex as a biological variable

**FIGURE 4:**
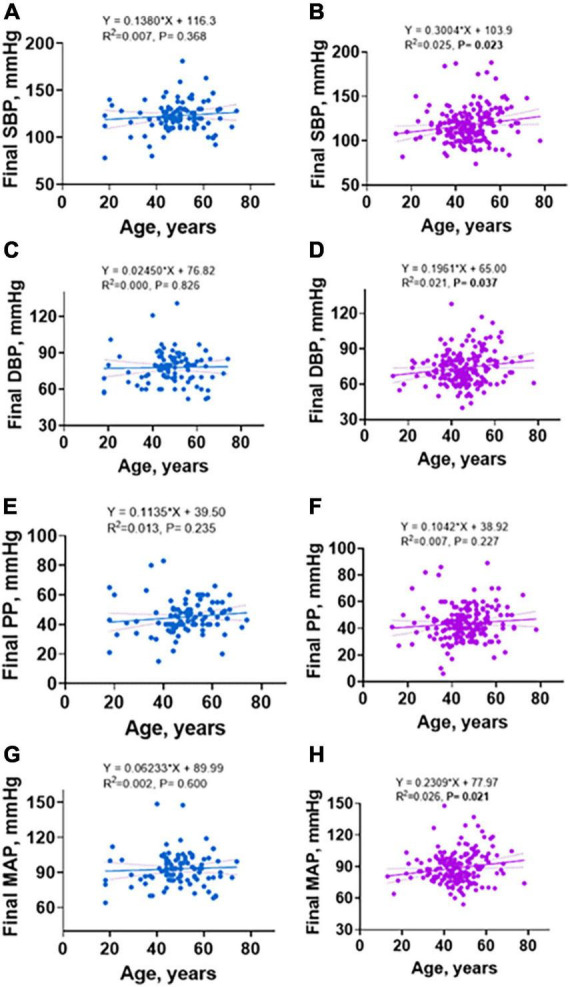
Linear regression of sex differences in relationship between age and final SBP, DBP, PP, and MAP. **(A)** SBP in males, **(B)** SBP in females, **(C)** DBP in males, **(D)** DBP in females, **(E)** PP in males, **(F)** PP in females, **(G)** MAP in males, **(H)** MAP in females.

Previous studies indicate that increasing age is associated with increased hypertension in the general population (17). In addition, women before menopause are protected from hypertension but this protection is lost after menopause (18, 19). While we did not screen women for menopause, we found that among men and women, blood pressure increased with age. However, the positive association between age and SBP, DBP, and MAP were only significant in women and not in men (Figures 4B,D,H, vs. Figures 4A,C,G, respectively) with no difference in PP for both sex (Figure 4F vs. Figure 4E). Consistent with this, in univariate and multivariate analysis, age was associated with increased risk for incident hypertension in the study (Table 3). Further, when segregated by sex, age was only associated with increased risk for incident hypertension in women on univariate analysis although it did not remain so on multivariate analysis (Table 4).

### Effect of antiretroviral therapy regimen and viral suppression on incident hypertension following initiation in men and women with HIV

At the end of the follow-up, the majority (*n* = 188, 61.2%) of the patients were still on NNRTIs, efavirenz- or nevirapine-based regimens; 22.1% (*n* = 68) and 16.6% (*n* = 51) switched to an INSTI DTG and protease inhibitors (PI) lopinavir boosted with ritonavir, respectively (Table 1). Among both males and females, a higher proportion of people who developed hypertension were on INSTI DTG-based (38.9%) regimen compared with the people who did not develop hypertension (19.9%), *p* = 0.004 (Table 1). The majority (84.9%, *n* = 203) were virally suppressed, and a higher proportion of virally suppressed participants was hypertensive (100%, *n* = 30) than normotensive (82.8%, *n* = 173), *p* = 0.011 (Table 1). In univariate analysis, switching to an INSTI or a PI was associated with an increased risk for incident hypertension (Table 3). In multivariable analysis, switching to a PI or INSTI remained significantly associated with incident hypertension. The risk for incident hypertension was 2–3-times higher for participants who switched to the INSTI (adjusted hazard ratio, aHR: 2.33, 95% CI 1.07–5.05; *p* = 0.032) or PI (aHR: 3.27, 95% CI 1.39–7.71; *p* = 0.007). We also found sex differences in ART use and risk for incident hypertension. On multivariate analysis, men were 16-times more likely to be hypertensive if they were on PIs (aHR: 3.27, 95% CI 1.72–156.6; *p* = 0.01) compared to the NNRTI-based regimen. Switching to either of the ART regimens was not significantly associated with incident hypertension in women.

### Incident hypertension following initiation of antiretroviral therapy and plasma creatinine in people living with HIV

**FIGURE 5:**
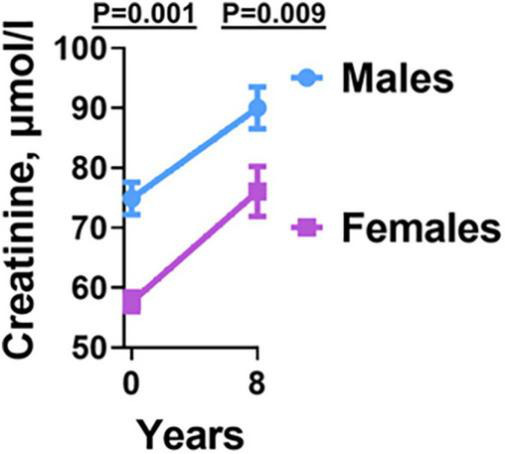
Creatinine levels before and after antiretroviral therapy in males and females. Males had higher Creatinine levels at both baseline and final year compared to females.

Previous studies have indicated that kidney disease is associated with hypertension in the general population (20, 21). To determine that incident hypertension in HIV is associated with kidney disease, we measured plasma creatinine at baseline and final year of follow-up. The final plasma creatinine level was higher in hypertensive compared with normotensive PLWH, *p* = 0.029 (Table 1). However, the values were within normal physiologic limits. Although both groups also had higher creatinine levels between baseline and final time-points, they were still within normal physiologic limits. Regardless of this, we found sex differences. Men had significantly higher creatinine values compared with women at both baseline and final year of follow-up (Figure 5).

### Sex differences in time course for development of incident hypertension following initiation of antiretroviral therapy in people living with HIV

**FIGURE 6:**
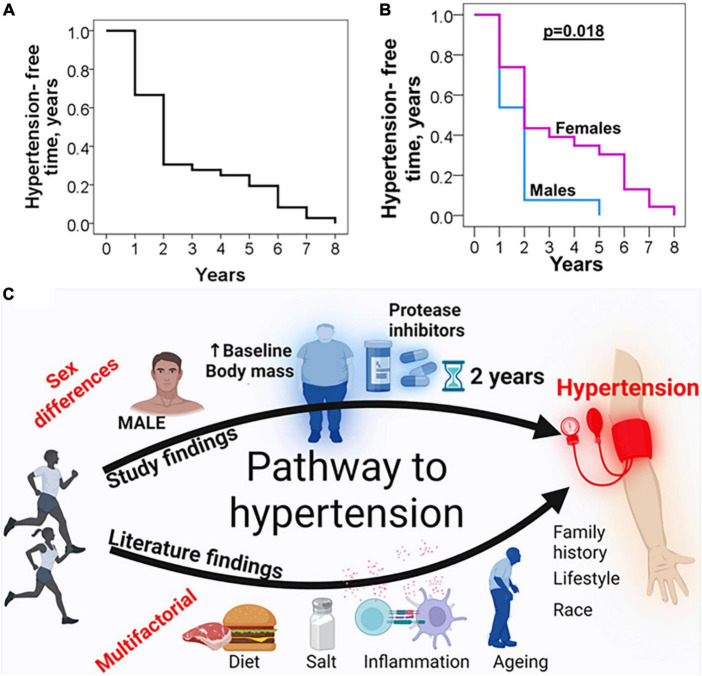
Sex differences in time course and predictors of incident hypertension. **(A)** The mean hypertension-free survival time for individuals who developed hypertension was 2.8 years. **(B)** Sex differences in time course to develop hypertension. Mean hypertension-free time for males was 2 years compared to 6 years for females. **(C)** Schematic of sex differences in predictors of hypertension (from our study) and general findings from literature on correlates of hypertension in the general population.

Previous studies indicate that in the general population, men develop hypertension earlier than females (22). We wanted to determine if this is the case in PWH. We found that the mean hypertension-free survival time for all participants who developed hypertension was 2.8 years including men and women (Figure 6A). We found that males developed hypertension earlier (2 years) when compared to females (6 years), (*p* = 0.018, Figure 6B).

To determine sex differences in factors associated with incident hypertension, we performed a multivariable analysis of all subjects, as well as among men and women. We found that among all PLWH, predictors of incident hypertension were the use of DTG or protease inhibitor, baseline MAP and SBP (Table 3). However, when stratified by sex (Tables 4, 5), only BMI and protease inhibitor use were significantly associated with incident hypertension in males (Table 5), whereas no factor remained significant in females (Table 4). In males, the adjusted hazard ratio for hypertension in those who used protease inhibitors was 16-times that of those on NNRTI/NRTI-based regimens (aHR: 16.45, 95% CI 1.72–156.64; *p* = 0.01).

## Discussion

Initiation of ART during HIV infection is associated with increased hypertension but the factors are not known. In this study, we demonstrated the time course and sex differences in predictors of incident hypertension in an adult population of 307 PLWH from Sub-Saharan Africa, Zambia, monitored retrospectively for 8 years. We found that higher baseline MAP and SBP were predictors of incident hypertension in PLWH albeit being in the normal range. We also found that switching to DTG - and lopinavir-based regimens was an additional risk factor for incident hypertension in all participants. Importantly, we found sex differences in the factors contributing to incident hypertension in PLWH. We found that the use of protease inhibitors and higher baseline BMI are predictors of incident hypertension in males but not in females. Our findings are summarized in Figure 6C and suggest the need to closely monitor blood pressure among PLWH being switched to newer ART regimens including protease inhibitors, especially among men who have higher BMI and baseline MAP and SBP.

Our findings indicate that the incidence of hypertension was 11.7%, which is lower than what has previously been reported in high-income countries (23–25) and other studies from Sub-Saharan Africa (26–28). The reason for the difference is not clear but could be related to other factors including diet and ethnicity as well as specific ART regimens. As expected, we found that increasing age was associated with incident hypertension as previously reported (26–28). Although baseline MAP, SBP, and DBP were significantly higher in those that developed hypertension when compared to those who remained normotensive, baseline MAP, and SBP but not DBP were predictors of incident hypertension on a multivariable analysis of all participants. In a previous study conducted among PLWH initiating ART in Uganda, SBP increased by 9.6 mmHg/year (95% CI 7.3–11.8) in the first 6 months of ART, then plateaued thereafter (29), however, MAP was not reported in this study. In our study, we observed that participants who developed hypertension did so a mean of 2.8 years after initiating ART. In the first year, about 33% of participants were hypertensive, a proportion that doubled by the end of the second year. This is similar to a study by Isa et al. where about 31% developed hypertension 12 months after initiating ART (30). However, this study did not follow PLWH beyond 12 months and thus, we cannot compare the time course of incident hypertension beyond the first year.

In this study, we found that the use of lopinavir/ritonavir or DTG was a predictor of incident hypertension; but nevirapine or efavirenz-based regimens were not. Although DTG and lopinavir have been associated with weight gain (10), dyslipidemia and/or lipodystrophy (31), respectively, the relationships between DTG, lopinavir/ritonavir, and incident hypertension are not well documented. However, in our previous study, the use of DTG was associated with metabolic syndrome, in which increased BP was a significant component (32).

Given the emerging evidence for sex differences in hypertension, we segregated our analyses by self-reported sex to determine sex-specific factors associated with hypertension on a multivariable regression model. We found that in males, the use of protease inhibitors and baseline BMI were significant predictors of hypertension but in females, no factor was associated with hypertension. This suggests that there could be specific factors that protect females from developing hypertension early when compared to males. Although it is beyond the scope of our study to ascertain the underlying mechanisms involved in this observed difference, several studies suggest a differential interplay of immune activation, hormones and the renin-angiotensin system among others that may play a significant role in sex related differences in the development of hypertension (8–22). Several animal studies have demonstrated that estrogen protects female mice from hypertension and even in the face of high-fat diets in both sexes (33, 34). It has been suggested that sex differences in diurnal sodium handling during diet-induced obesity in rats may be the underlying mechanism for the sex differences in the development of salt-sensitive hypertension (35). This may likely explain why in our study females were still protected from hypertension even though their BMI was higher than that of males throughout the follow-up period. It is well known in the general population that blood pressure is higher in males than in females at similar ages but after menopause, females’ blood pressure increases to levels higher than that of males (36). This has also been confirmed in PLWH by the largest available study; the Data Collection on Adverse Events of Anti-HIV Drugs (D:A:D) study (37). Apart from estrogen being the main protector of hypertension in premenopausal women, androgens have also been implicated to play a role since hormone replacement with estrogen in postmenopausal women does not significantly reduce blood pressure (36). Another important dimension and emerging understanding of sex differences in hypertension was reported by Ji et al. (38). Ji et al. studied trajectories of blood pressure elevation examined serially over four decades in 32 833 individuals (54% females) with age spanning from 5 to 98 years. They found that females actually had a steeper increase in blood pressure compared to males throughout the life course, beginning in the third decade and this trend, predisposes women to CVDs later in life that tend to present differently in women compared with men (38).

As regards the use of protease inhibitors, previous studies have reported that protease inhibitors increase the risk of developing hypertension in people with HIV (6, 39). Particularly the use of Lopinavir/ritonavir was significantly associated with an increased incidence of new-onset hypertension (6). However, there is a scarcity of studies focused on sex differences in specific ART regimens including protease inhibitors in contributing to the onset of hypertension.

An interesting finding in our study is that while CD4 T cell counts increased following initiation of ART as did blood pressure, there was no significant correlation between them, and we did not observe any sex differences. Our previous studies and those of others focused on the role of immune cell activation in HIV have indicated that immune reconstitution early in ART may be responsible for high blood pressure and hypertension due to immune activation (2, 4, 15). Reis et al. studied BP modulation in a group examined from pre-ART through the first 2 years of ART (15). They found that immune-suppressed PLWH had a significantly lower BP before initiating ART but during the first 2 years of ART they had a significantly greater increase in BP compared to the pre-ART period. They also found that lower BP before ART initiation and greater increases in BP were strongly positively associated with CD4 + T-cell counts. However, the sex difference was not determined in this study. Although we did not find significant differences in CD4 counts between hypertensive and normotensive participants in our current study, our previous studies indicated that CD4 count is a predictor of hypertension (2). Further detailed research is needed to understand the role of immune cells in the development of hypertension in PLWH.

Limitations of our study include a lack of a healthy control group of HIV-uninfected persons as well as a relatively small number of participants compared to other previously reported studies. The limitation in sample size was as a result of a lot of missing data on the outcome variable for many participants that we had to exclude. Also, we did not determine the specific time point participants switched to INSTIs and protease inhibitors to examine a relationship with incident hypertension. This is especially important because previous studies have indicated the role of protease inhibitors in hypertension (6–39). Further investigations are required to understand the relationships between INSTIs, protease inhibitors and the development of hypertension in PLWH. Another limitation is that we did not screen for menopausal status to determine the contribution of menopause to the risk for hypertension in women. These data would have provided more information for us to explore the sex differences relating to estrogen hormones. Finally, the potential of unmeasured confounder role of social determinants of health such as economic, social, environmental, and psychosocial factors that significantly contribute to hypertension and CVD (40) could not be determined due to the nature of our study.

In conclusion, we found that PLWH who develop hypertension are likely to do so within 2 years for males and 6 years for females after initiating ART. Age, baseline MAP, baseline systolic blood pressure, and use of protease inhibitor- or INSTI- based ART regimens were significant predictors of incident hypertension in this specific population. However, in males but not in females, the use of protease inhibitors and high baseline BMI were the only significant predictors of incident hypertension. Thus, it is critical to closely monitor blood pressure in the first 2 years following ART initiation to mitigate incident hypertension, especially for PLWH with these identified risk factors.

## Data availability statement

The original contributions presented in this study are included in the article/supplementary material, further inquiries can be directed to the corresponding author.

## Ethics statement

This study was approved by the University of Zambia Biomedical Research Ethics Committee (IRB00001131 of IORG0000774) and the National Health Research Ethics Board (NHREB) under Reference number: 981-2020. All participants signed a consent form before being included in the study. The patients/participants provided their written informed consent to participate in this study.

## Author contributions

SKM conceived the study, drafted the manuscript, and conducted the data analysis. SN, DH, AK, and SMM revised the study concept and supervised data collection. SKM and BH conducted the data collection. BH and DH interpreted all statistical aspects relating to regression analysis. KM, JP, SN, AK, SMM, and KP made substantial revisions to the whole manuscript. All authors read and approved the final manuscript.

## Abbreviations

ASR: adjusted standardized ratio
BMI: body mass index
BP: blood pressure
CVD: cardiovascular disease
DBP: diastolic blood pressure
DTG: dolutegravir
HR: hazard ratio
aHR: adjusted hazard ratio
IQR: interquartile range
INSTI: integrase strand transfer inhibitor
PI: protease inhibitors
LPV/r: ritonavir-boosted lopinavir
MAP: mean arterial pressure
NHREB: National Health Research Ethics Board
NNRTI: non-nucleoside reverse transcriptase inhibitor
NRTI: nucleoside reverse transcriptase inhibitor
PLWH: persons living with HIV
REDCap: Research Electronic Data Capture
SBP: systolic blood pressure
SSA: sub-Saharan Africa
SPSS: statistical package for social sciences.
